# Therapeutic Potential of Select Dietary Compounds in the Management of Hypertension and its Cardiovascular Complications

**DOI:** 10.3390/molecules27217222

**Published:** 2022-10-25

**Authors:** Aleena Francis Valookaran, Jenny Bouchard, Basma Milad Aloud, Sijo Joseph Thandapilly, Thomas Netticadan

**Affiliations:** 1 Morden Research and Development Centre, Agriculture and Agri-Food Canada, Morden, MB R6M 1Y5, Canada; 2 Canadian Centre for Agri-Food Research in Health and Medicine, Winnipeg, MB R2H 2A6, Canada; 3 Richardson Center for Functional Foods and Nutraceuticals, Winnipeg, MB R3T 2N2, Canada; 4 Department of Food and Human Nutritional Sciences, University of Manitoba, Winnipeg, MB R3T 2N2, Canada; 5 College of Pharmacy, University of Manitoba, Winnipeg, MB R3E 0T5, Canada; 6 Department of Physiology and Pathophysiology, University of Manitoba, Winnipeg, MB R3E 0J9, Canada

**Keywords:** resveratrol, coenzyme Q10, quercetin, docosahexaenoic acid, eicosapentaenoic acid, bioactive compounds, hypertension

## Abstract

Hypertension is a common risk factor for cardiovascular disease and mortality worldwide. Proper nutrition and diet are known to play an indispensable role in the treatment and management of hypertension. Bioactive compounds that occur in small quantities in foods such as onions, fish and red wine are being intensively studied to uncover their vasoprotective, antioxidant, anti-proliferative and anti-inflammatory effects which are beneficial to attenuate chronic disease and protect human health. In this article, the anti-hypertensive, and cardio-protective effects of five food-derived bioactive compounds: resveratrol, quercetin, coenzyme Q10, DHA and EPA and their proposed mechanisms of action are reviewed in detail.

## 1. Introduction

High blood pressure, also known as hypertension, is a chronic condition affecting over 1.28 billion people globally and results in nearly 9.4 million deaths every year due to the associated complications [1,2]. Current therapeutic interventions have indisputably shown great promise in patients with hypertension, yet, about 30% of the patients are still struggling to manage their elevated blood pressure, irrespective of their compliance [3].

In this context, over the years, several food-derived bioactive constituents including angiotensin-converting enzyme (ACE) inhibitory peptides, vitamins C and E, flavonoids, flavanols, cathecins, anthocyanins, phenolic acids, polyphenols, tannins, resveratrol, polysaccharides, fibre, saponin, and sterols have been evaluated for their anti-hypertensive efficacy in various experimental settings [4]. Although several compounds have shown potential benefits in in vitro and pre-clinical animal studies, only limited bioactive compounds have shown blood pressure-lowering efficacy in randomized human feeding studies.

**Figure 1:**
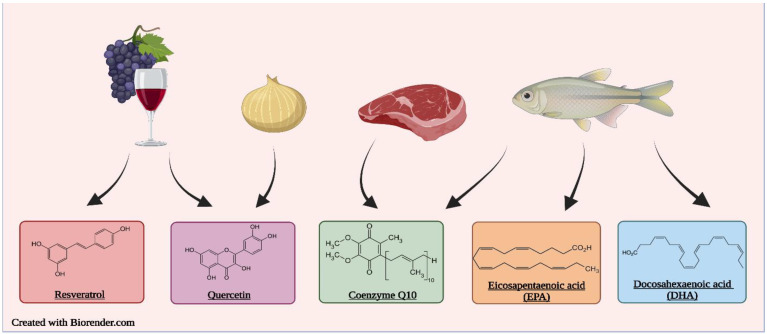
Most popular food sources of the bioactive compounds are resveratrol, quercetin, coenzyme Q10, EPA, DHA.

In this review, we provide a detailed insight into the current research developments on bioactive compounds for the management and treatment of hypertension. By providing a brief overview of the pathogenesis of hypertension, we aim to review the anti-hypertensive effects of five selected food-derived bio-actives, resveratrol, quercetin, coenzyme Q10, docosahexaenoic acid (DHA) and eicosapentaenoic acid (EPA), which have been shown efficacy in randomized human feeding studies and try to elucidate the modes of action (Figure 1). We further deliberate on the challenges and future perspectives of these studies and its findings.

## 2. Literature Search

The literature search was conducted with the use of the scientific databases and search engines Pubmed, Google Scholar and Scopus. The search terms “resveratrol”, “docosahexaenoic acid”, “DHA”, “eicosapentaenoic acid”, “EPA”, “quercetin” and “coenzyme Q10” were used in combination with any of the following terms: “hypertension”, “blood pressure” “spontaneously hypertensive rat”, “SHR”, “renovascular hypertension” “2K1C”, “pulmonary hypertension”, “salt sensitive hypertension”, “Dahl salt sensitive rat”, “clinical trial”. There were no limitations placed on the literature search pertaining to the publication date of the studies. Case reports, commentaries and editorials were excluded from the literature search. For the preclinical trials, the studies were only considered if the animal model fell under the following categories: pulmonary hypertension, spontaneously hypertensive rats, renovascular hypertension and salt sensitive hypertension. Normotensive animal models were excluded from the literature search. For the clinical trials, any studies evaluating the select bioactive compounds in combination with other treatments were excluded. Studies pertaining to metabolites or analogues of the compounds were also excluded. Furthermore, only clinical trials containing hypertensive subjects were considered.

## 3. Hypertension

Hypertension or high blood pressure is among the most prevalent and important modifiable risk factors for cardiovascular disease and premature mortality [5]. As of 2021, the World Health Organization estimates that 1.28 billion adults aged 30–79 years have hypertension, two-thirds of which live in low- and middle- income countries [6]; this number may be higher, as approximately 46% of adults with hypertension are unaware of their condition [6]. In Canada, roughly 25% of the adult population are living with hypertension and the lifetime incidence of developing hypertension is approximated to be 90% [7]. Furthermore, hypertension continues to be a leading cause of premature death worldwide accounting for 8.5 million deaths annually [8]; this is why hypertension is colloquially known as the “silent killer”.

The 2017 American Heart Associate guidelines define hypertension or systemic arterial hypertension as a chronic increase in systolic blood pressure (SBP) above 130 mm Hg and/or diastolic blood pressure (DBP) above 80 mm Hg measured in a relaxed sitting posture [9]. In addition, blood pressure classification in adults is as follows: normal (<120 mm Hg SBP and <80 mm Hg DBP), prehypertensive (120–129 mm Hg SBP and <80 mm Hg DBP), stage 1 hypertension (130–139 mm Hg SBP or 80–89 mm Hg DBP), and stage 2 hypertension (≥140 mm Hg SBP or ≥90 mm Hg DBP) [9]. Despite the major health, social, and financial impact of hypertension, it often remains undiagnosed and/or ineffectively treated [10]. Several factors have been implicated in the development of hypertension. Modifiable factors include smoking, obesity, a sedentary lifestyle, high saturated and trans-fat consumption, high sodium intake, low potassium, and calcium intake. Non-modifiable risk factors are genetic predisposition, age, sex, and ethnicity [6].

Regulation of blood pressure is a complex integrated response involving a variety of organ systems, including the central nervous system, cardiovascular system, kidneys, and adrenal glands. Uncontrolled hypertension can damage these organs, and it is a major contributor to the development of congestive heart failure, end-stage renal disease, and stroke [10]. Hypertensive heart disease is one of the major complications of untreated hypertension. The cardiac consequences of high blood pressure include abnormalities in the structure and function of the myocardium, including left ventricular hypertrophy, systolic and diastolic dysfunction, and in severe cases, overt heart failure [10]. It has been reported that the lifetime risk for developing heart failure is 1 in 9 men and 1 in 6 women among patients with hypertension and without an established myocardial infarction, highlighting the risk conferred by hypertension alone [11]. Although the exact molecular mechanisms by which hypertension predisposes patients to heart failure have not been fully elucidated, it is generally believed that chronic blood pressure elevation leads to the development of left ventricular hypertrophy, a compensatory response by which the heart walls increase in size in an attempt to counter the increased ventricular systolic wall stress [12]. At the cellular level, the development of cardiac hypertrophy involves an increase in cardiomyocyte size, enhanced protein synthesis, and sarcomeric reorganization [13]. Prolonged cardiac hypertrophy becomes maladaptive, and it is associated with a rapid decline in the contractile performance of the myocardium (systolic and diastolic dysfunction), eventually leading to heart failure [10]. While the development of hypertension has been attributed to aberrations in vasculature, central nervous system, and kidneys, accumulating evidence suggests that immune system dysregulation also contributes. Cells of the innate and adaptive immune systems have been reported to play important roles in the initiation and maintenance of hypertension in different animal models. For example, studies have shown that mice lacking adaptive immune cells are resistant to blood pressure elevation in response to angiotensin II and high salt [14]. Hypertension is characterized by enhanced expression of adhesion molecules on blood vessels, the heart, and the kidneys; therefore, enhancing extravasation and accumulation of immune cells such as macrophages and T lymphocytes in these organs; these infiltrating cells secrete and stimulate other immune cells to secrete pro-hypertensive cytokines such as interleukin 6 (IL-6), IL-17 and tumor necrosis factor-α (TNF-α) [14,15]. Therefore, assessment of immune cell function in hypertension is crucial in the discovery and mechanistic understanding of novel therapeutic strategies to prevent and treat hypertension.

The established anti-hypertensive drug classes available for treating hypertension/heart failure include diuretics, β-adrenergic receptor blockers, calcium channel blockers, angiotensin-converting enzyme inhibitors, angiotensin receptor blockers, and aldosterone antagonists [16]. However, many of these medications have side effects that may adversely impact the quality of life. Consequently, new effective and safer therapeutic agents for hypertension and hypertensive heart disease need to be developed to improve the quality of life in hypertensive patients. Observational trials have reported an increased risk of cardiovascular disease as SBP and DBP increase [17]. In a meta-analysis of 61 prospective trials, every 20/10 mm Hg increase in blood pressure was correlated with a doubling of the risk of mortality from heart disease and cerebrovascular accident [9,18]. It is important to recognize that the lifetime risk of developing hypertension is three times higher in the prehypertensive population as compared with normotensive subjects [19]. Therefore, it is essential to detect and manage elevated blood pressure at early stages to reduce the risk of cardiovascular comorbidities and premature death.

Hypertension is classified as either idiopathic/essential (primary) or non-essential (secondary) hypertension [20,21,22]. Essential hypertension affects more than 90% of hypertensive patients and is a heterogenous disorder of genetic origin influenced by different environmental factors [20]. In contrast, non-essential hypertension is usually caused by a specific secondary condition such as renal or endocrine disorders [21]. Several interrelated contributing factors have been reported to be involved in the pathogenesis of essential hypertension, including genetic predisposition, enhanced sympathetic nervous system activity, increased peripheral vascular resistance, endothelial dysfunction, and imbalance in the renin-angiotensin-aldosterone system [20]. Non-essential hypertension is the direct result of a specific disorder such as renal parenchymal disease (chronic kidney disease); renovascular disease including fibromuscular dysplasia and atherosclerotic renal artery stenosis; as well as endocrine abnormalities such as primary aldosteronism, pheochromocytoma, high cortisol levels, and thyroid or parathyroid abnormalities [21].

### 3.1. Regulation of Blood Pressure

Blood pressure regulation is defined as the control of blood supply to a particular organ to match its metabolic needs [23]. The main cardiovascular factors that determine blood pressure are cardiac output and total peripheral resistance, which are mainly influenced by neurohormonal mechanisms and intravascular blood volume [24] Control of blood pressure involves different complex mechanisms, including neural mechanisms, renal-endocrine mechanisms, and endothelium-dependent mechanisms [23,24]. Imbalance or dysregulation of blood pressure control mechanisms can lead to chronic blood pressure elevation, which will eventually lead to target organ damage, including overt heart failure, end-stage renal disease, and stroke [10,24]. Blood pressure control mechanisms can be classified into local and global regulation mechanisms. Local mechanisms regulate blood pressure by controlling blood supply to body tissues acutely through vasoconstriction and vasodilation, and chronically by modifying the number and diameter of blood vessels supplying body organs [23]. Besides local blood pressure control mechanisms, global control mechanisms regulate blood flow by modifying cardiac output and control blood pressure mainly by regulation of the sympathetic nervous system. The renal-endocrine system is another crucial chronic control mechanism that regulates blood pressure by regulating sodium and fluid homeostasis [23].

#### 3.1.1. Sympathetic Nervous System

An increase in the activity of the sympathetic nervous system (SNS) is associated with higher cardiac output, tachycardia, higher norepinephrine levels, and peripheral vasoconstriction in hypertensive young adults [23]. The SNS is also more active in hypertensive patients with prediabetes, sleep apnea, heart disease, and kidney disease [23,24]. Furthermore, studies have revealed that the occurrence and maintenance of essential hypertension are related to an imbalance in the autonomic nervous system with increased stimulation of the SNS and decreased parasympathetic nervous system stimulation [24]. Baroreceptors, pressure changes sensing receptors, are located in different places in the arterial system, but their main locations are the carotid sinus and the aortic arch. An acute rise in blood pressure causes the carotid artery to stretch and stimulate baroreceptors to send impulses to the central nervous system to decrease the activity of the SNS [23,24]. SNS also plays an important role as a chronic blood pressure regulator by stimulating renin release in the juxtaglomerular apparatus in the kidney by activating the sympathetic renal nerve [23]. Enhanced sympathetic renal nerve activity with associated salt retention and sustained hypertension have been reported in obesity-induced hypertension models [25].

#### 3.1.2. Renin-Angiotensin-Aldosterone System

The renin-angiotensin-aldosterone system (RAAS) is a hormonal system that plays a fundamental role in blood pressure regulation by controlling vasoconstriction and sodium and fluid homeostasis, and it is an important mediator of essential hypertension [24]. The most important function of the RAAS is to maintain pressure-fluid homeostasis in the renal system by preserving adequate renal perfusion in cases of extracellular fluid depletion as a consequence of increased sodium and fluid excretion. To maintain pressure-volume balance, when the volume of the extracellular fluid expands, the activity of the RAAS is suppressed to enhance sodium and water excretion [24]. Renin, also referred to as angiotensinogenase, is an aspartic protease enzyme that is synthesized in the juxtaglomerular apparatus in the kidneys. Upon stimulation with different stimuli including stimulation of the renal sympathetic nerve, and vasodilation, renin is released and activates the hydrolysis of angiotensinogen (a protein that is synthesized and released to the systemic circulation by the liver) to angiotensin I [24]. Angiotensin I is a mild vasoconstrictor, and it is not strong enough to induce major circulatory changes [23]. ACE is a key mediator in the RAAS that regulates blood pressure by converting angiotensin I to the potent vasoconstrictor angiotensin II, which plays a central role in the pathogenesis of essential hypertension. Angiotensin II mediates its actions by binding to and activating the transmembrane G protein-coupled receptors (AT1 and AT2); it activates renal sodium reabsorption, induces endothelial dysfunction, and has proinflammatory properties; therefore, angiotensin II is implicated in the pathogenesis of hypertension-related microvascular (nephropathy and retinopathy) and macrovascular damages (heart attacks and cerebrovascular accidents) [24]. In addition to ACE, ACE2 has a crucial role in blood pressure regulation by mediating the conversion of angiotensin II to angiotensin (1–7). Angiotensin (1–7) stimulates vasodilation, enhances sodium and fluid excretion, and has been reported to have cardiovascular protective activities [24]. Aldosterone is a mineralocorticoid hormone produced primarily by the adrenal cortex in the adrenal gland that regulates blood pressure by enhancing renal sodium reabsorption via activating renal epithelial sodium channels in the collecting tubules [23,24]. Aldosterone contributes to the development of hypertension by enhancing vascular extracellular matrix accumulation, endothelial dysfunction, and oxidative stress [24].

#### 3.1.3. Endothelium

The endothelium plays a crucial role in the control of blood pressure by regulating blood vessel tone through nitric oxide production (also referred to as endothelium-derived relaxing factor). In response to blood flow-induced shear stress, nitric oxide is synthesized by endothelial cells from L-arginine by the action of nitric oxide synthase. Nitric oxide induces vasodilatation by stimulating guanylyl cyclase to produce cyclic guanosine monophosphate, which mediates vascular smooth muscle relaxation. Studies have demonstrated that hypertensive patients have lower levels of nitric oxide compared with their normotensive counterparts [24]. Furthermore, a decrease in nitric oxide levels has been reported to potentiate the vasoconstrictor actions of angiotensin II [23]. In addition to nitric oxide, endothelial cells produce the potent vasoconstrictor polypeptide, endothelin 1 (ET1). By binding to ETA receptors located in the vascular smooth muscle cells, ET1 induces systemic vasoconstriction resulting in an increase in arterial blood pressure. ET1 can also elevate blood pressure by enhancing the SNS [23]. Even though results from different studies have not consistently shown increases in ET1 levels in hypertension, studies have reported that hypertensive patients have a higher sensitivity to the vascular effects of ET1 [24]. Endothelial dysfunction strongly contributes to the development of hypertension. In addition to pressure-related vascular damage, studies have demonstrated that oxidative stress contributes to the development of endothelial dysfunction in hypertension [24]. Increased superoxide anions levels as a result of decreased superoxide dismutase activity reduces the bioavailability of nitric oxide by binding to it and forming peroxynitrite, a highly reactive oxidant.

### 3.2. Hypertensive Heart Disease

Several prospective cohort studies have revealed that high blood pressure increases the risk of premature mortality and cardiovascular complications [26]. Hypertensive subjects have more than twice the risk for developing ischemic heart disease, and more than triple the risk for developing congestive heart failure and cerebrovascular accident [27]. Several forms of heart disease including left ventricular hypertrophy, coronary heart disease, heart failure and sudden cardiac death have been etiologically linked to hypertension [26].

#### 3.2.1. Left Ventricular Hypertrophy

Left ventricular hypertrophy (LVH) is defined as the enlargement and thickening of the left ventricle of the myocardium. Based on echocardiographic measurements, LVH is diagnosed when left ventricular weight indexed to body surface area is ˃131 g·m^−2^ for men and ˃100 g·m^−2^ for women. The risk of developing LVH in normotensive subjects has been estimated to be 1.3%–1.6%, whereas the risk increases in subjects with mild and severe hypertension to 2.7%–5.6% and 75.6–82.6%, respectively [27]. LVH is associated with higher mortality rates for ischemic heart disease, heart failure, and cerebrovascular accidents. The Framingham heart study demonstrated that hypertensive subjects with LVH have a poor prognosis irrespective of blood pressure levels [26,27]. LVH is also linked to a higher risk of cerebrovascular accident, myocardial infarction, and peripheral arterial disease [20]. Ventricular enlargement can be classified into concentric or eccentric LVH. While in concentric LVH the walls of the left ventricle thicken relative to the internal cavity, eccentric LVH mainly involves the enlargement of the intraventricular septum. Concentric LVH is commonly seen in patients with mildly to severely elevated blood pressure and is associated with normal or decreased cardiac output [27].

Several etiological factors have been implicated in the pathogenesis of LVH in hypertension, including pressure overload with an increase in total peripheral resistance, as well as neurogenic and hormonal factors [27].

##### Left Ventricular Pressure Overload

The main determinants of pressure overload of the left ventricle in the context of hypertension are aortic stenosis and increased arterial blood pressure. Both increased intraventricular pressure and aortic stenosis result in cardiomyocyte hypertrophy and enhanced peri-myocytic extracellular matrix deposition [27].

##### Renin-Angiotensin-Aldosterone-System (RAAS)

Different hypertrophic factors, including the components of the RAAS, such as angiotensin-II and aldosterone have an important role in the development of hypertensive heart disease by inducing cardiac hypertrophy [28]. Experimental data suggest that the stimulation of the RAAS by suprarenal aortic constriction or intravenous administration of angiotensin-II or aldosterone can directly induce cardiac hypertrophy and fibrosis. The involvement of the RAAS in the development of LVH in hypertension is evidenced by the efficacy of the angiotensin-converting enzyme inhibitors and angiotensin-II receptor blockers in preventing/regressing hypertensive LVH [27].

##### Aldosterone

Besides its blood pressure-elevating effects, such as enhancing sodium retention, and stimulating the SNS, aldosterone has been reported to induce cardiac fibrosis and potentiate the fibrotic effects of angiotensin-II in hypertension. Therefore, aldosterone has a critical role in the pathogenesis of hypertension-associated LVH; it has also been suggested that the effects of aldosterone with regards to the pathogenesis of hypertensive heart disease and heart failure are independent of renin and angiotensin-II because the administration of an aldosterone receptor antagonist, Aldactone, in combination with other standard heart failure medications markedly improved the outcomes in individuals with severe heart failure [27].

##### Sympathetic Nervous System

Activation of the SNS in hypertension may contribute to the development of LVH. The role of SNS in stimulating LVH is evidenced by the presence of LVH and heart failure in subjects with pheochromocytoma, a catecholamine-secreting tumor of the medulla of the adrenal gland [27].

##### High Sodium Intake and Salt Sensitivity

Another important factor that can affect left ventricular mass in hypertension is high dietary sodium consumption. In spontaneously hypertensive rats the development of LVH was potentiated by a high-sodium diet. Furthermore, high-sodium intake caused an increase in the myocardial weight in Wistar Kyoto Rats independently of blood pressure [27,29]. Salt sensitivity is known to increase cardiac output by promoting an expansion of extracellular fluid volume, increasing SNS activity, impairing the RAAS, and decreasing NO synthesis in the endothelium resulting in increased vascular resistance [30]. Salt sensitivity has been identified as a critical determinant of the influence of dietary sodium on LVH since hypertensive patients with salt sensitivity are more likely to develop LVH compared with salt-resistant hypertensive patients [31].

##### Renovascular Hypertension

Renovascular hypertension is defined as high blood pressure as a result of the narrowing of the arteries in the kidneys; it is a major form of secondary hypertension that involves decreased blood flow to the kidneys and increased activation of the RAAS [32]; it accounts for 1–5% of all cases of hypertension [33]. The most common causes of renovascular hypertension are atherosclerotic renal artery stenosis, fibromuscular dysplasia, compression, dissection, or infarction of the renal artery [34]. In addition to vasoconstriction and sodium retention, increased activity of the RAAS axis leads to activation of inflammatory and fibrogenic mechanisms, which result in vascular remodeling, renal tissue fibrosis and LVH. Increased sympathetic activation, oxidative stress and endothelial dysfunction are associated with increased activity of RAAS and contribute to the development of renovascular hypertension [33].

##### Pulmonary Hypertension

Pulmonary arterial hypertension (PAH) is a progressive disease associated with increased construction and remodeling of pulmonary arteries causing increased pulmonary vascular resistance, right ventricular hypertrophy and dysfunction and heart failure [35] It is characterized by resistance to apoptosis, increased proliferation, and migration of pulmonary artery smooth muscle cells (PASMCs) [36]. PAH is defined by a mean pulmonary arterial pressure (mPAP) of ≥25 mmHg in a relaxed sitting position, a pulmonary vascular resistance of >3 Wood units and an end-expiratory pulmonary artery wedge pressure of ≤15 mmHg [37]. It is estimated that globally, PAH affects 1% of the population. Though the symptoms are often unspecific, PAH is often accompanied by exercise intolerance, extreme fatigue and exhaustion and syncope after slight exertion. In the case of cardiac decompensation, cervical venous congestion, ascites, and edema occur due to the rise of right cardiac filling pressures [38]. Though PAH is associated with RV dysfunction, it is often a consequence of left-sided heart disease and is usually a result of systemic hypertension and ischemic heart disease [39].

### 3.3. Food-Derived Bioactive Compounds for Management of Hypertension

**Figure 2:**
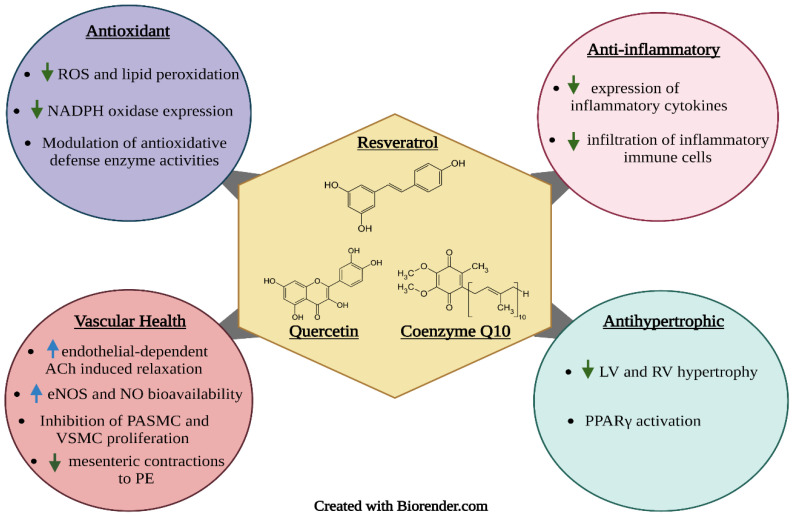
Proposed mechanisms of action of the bioactive phenolic compounds resveratrol, quercetin, and coenzyme Q10. Upward facing arrows indicate an increase in activity, while downward facing arrows indicate a decrease in activity.

**Figure 3:**
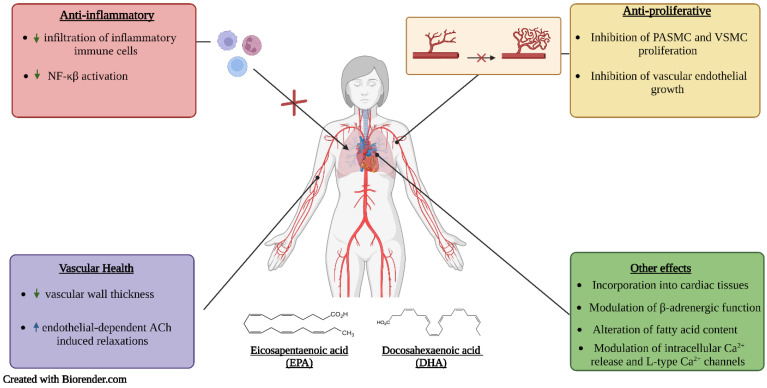
Proposed mechanisms of action of EPA and DHA. Upward facing arrows indicate an increase in activity, while downward facing arrows indicate a decrease in activity.

Proper nutrition is imperative in the prevention and management of hypertension. Poor dietary habits, such as the consumption of high amounts of sodium and saturated and trans fats and low amounts of fibre, are considered important risk factors for hypertension. One important strategy is the use of functional foods and nutraceuticals; these are foods that offer health-related benefits and are considered advantageous for the treatment and prevention of disease [40]. This review emphasizes the importance of frequent consumption of healthy foods that contain bioactive compounds such as phenolic antioxidants (resveratrol, quercetin, and coenzyme Q10) (Figure 2) and essential fatty acids (DHA and EPA) (Figure 3), which exhibit antioxidant, anti-hyperlipidemic, anti-inflammatory and anti-proliferative effects that confer protection against hypertension and aid in the management of the condition.

## 4. Quercetin

Quercetin is a bitter tasting flavonoid known for its potent antiviral, anti-carcinogenic, antioxidant, anti-inflammatory and disease-prevention abilities [41,42,43,44]. Structurally, quercetin is a pentahydroxyflavone containing five hydroxy groups located at the 3-, 3’-, 4’-, 5- and 7-positions [45]; it is known to be one of the most abundant flavonoids in fruit, vegetables, and red wine. Sources of quercetin include onions, shallots, brassica vegetables, apples, grapes, berries, tomatoes honey, tea, nuts, and seeds [42,44]. Numerous studies have determined the health advantages of quercetin, including the capacity to prevent cardiovascular disease by lowering blood pressure.

### 4.1. Preclinical Trials

#### 4.1.1. Pulmonary Hypertension Models

Quercetin has been reported to exert protective effects against PAH such as decreased mPAP, prevention of right ventricular hypertrophy and remodeling of pulmonary arteries in various PAH animal studies [36,46,47]. In addition, in monocrotaline (MCT) induced PAH rat models, quercetin supplementation was found to decrease proliferating cell nuclear antigen expression and wall thickness and area of pulmonary arteries [46]; it inhibited a decrease in K_V_ currents and the overexpression of 5-HT_2A_ and inducible nitric oxide synthase (NOS) induced by MCT in PASMCs while also reducing AKT and S6 phosphorylation [47]. One study found that quercetin attenuated MCT-induced expression of inflammatory cytokines HIF-1, ET-1, transforming growth factor β1 (TGFβ1), Vascular endothelial growth factor, IL-1, IL-6 and TNF-α in lung tissues. Furthermore, it significantly increased hepatocyte growth factor and N-acetylcysteine levels, which respectively play roles in cell proliferation and apoptosis inhibition [48]. Quercetin was shown to also inhibit PASMC proliferation by modulating the expression of various functional proteins that are related to the growth and metastasis pathways of PASMCs in a chronic hypoxia model of PAH; this included the inhibition of the TrkA/AKT signaling pathway, which resulted in decreased migration of PASMCs, cell cycle arrest and apoptosis induction [36].

#### 4.1.2. Renovascular Hypertension Model

The two-kidney, one-clip Goldblatt hypertension animal model (2K1C) is commonly used to study renovascular hypertension, as decreased perfusion to the kidneys through partial obstruction of the renal artery will persistently increase blood pressure [32]. Quercetin has been shown to possess anti-hypertensive properties in this model by the following studies. Choi et al. reported that quercetin treatments augmented aortic acetylcholine-induced relaxation and inhibited aortic phenylephrine-induced contraction [49]. Garcia-Saura et al. demonstrated that quercetin treatment reduced systolic blood pressure (SBP), endothelial dysfunction, cardiac hypertrophy, and proteinuria in this model [32]. Pereira et al. concluded that quercetin significantly reduced vascular Nicotinamide adenine dinucleotide phosphate (NAD(P)H) oxidase activity, reactive oxygen species (ROS) levels and metalloproteinase-2 activity. Oxidative stress and increased activity of NAD(P)H oxidase play a role in endothelial dysfunction, hypertrophy and hypertension-induced arterial contractility [50]. Excessive ROS concentrations in vascular smooth muscle cells (VSMC), including superoxide radicals produced by NAD(P)H oxidase, activate proliferative signaling pathways, which result in vascular cell proliferation and remodeling. ROS also modulates gene expression and activity of metalloproteinases that cleave extra- and intracellular proteins, which contribute further to vascular remodeling and changes in function [50]. Montenegro et al. reported that quercetin treatment effectively reduced SBP, NADPH oxidase activity and vascular superoxide production while improving both endothelial-dependent responses to acetylcholine and plasma nitrite and nitroso species concentrations [51]. Plasma nitrite and nitroso compounds have been proven to be relevant markers of nitric oxide (NO) formation. NO may be scavenged by superoxide anions, reducing its bioavailability, and therefore diminishing its vasodilating properties. The results of these studies suggest that quercetin is able to improve endothelial function and increase NO formation through potent antioxidant effects in the 2K1C animal model.

#### 4.1.3. SHR Model

The spontaneously hypertensive rat (SHR) model is an animal model used to study primary hypertension; it is the most studied model of hypertension to date. In SHRs, hypertension develops around 5–6 weeks of age. In the adult phase, systolic blood pressure can reach 180–200 mmHg [52]. SHRs eventually develop characteristics of cardiovascular disease such as increased oxidative stress, cardiac hypertrophy, vascular dysfunction, and ultimately progress to heart failure at 18–24 months [53].

Several studies have evaluated the cardioprotective effects of quercetin in an SHR model. Quercetin and its metabolites effectively reduce an increase in blood pressure and heart rate [54,55,56,57,58,59,60,61,62,63,64,65,66] and improve left ventricular [62,65,66] and renal hypertrophy [62]; it enhances the endothelium-dependent aortic vasodilation induced by acetylcholine [54,57,59,62,63,67,68], but had no effect on the endothelium-independent response induced by nitroprusside [54,63,68]. Quercetin also reduces mesenteric contractions in response to phenylephrine, which is associated with depolarization and an increase in smooth muscle intracellular calcium concentration [54,57,58,68,69]; these results suggest that this flavonoid is able to provide vascular protection by ameliorating endothelial dysfunction in a hypertensive model.

Quercetin possesses potent antioxidative properties that allow it to effectively attenuate oxidative stress in this model. It is reported that dietary quercetin improves oxidation status in many ways; it attenuates lipid peroxidation by reducing both plasma and hepatic malondialdehyde (MDA) levels [55,60,62,64]. MDA is a final product in the peroxidation of polyunsaturated fatty acids and is commonly used as a marker for oxidative stress. Furthermore, quercetin treatment significantly increased glutathione peroxidase activity and reduced aortic superoxide production in SHRs [55,62]. The flavonoid is also able to prevent vascular oxidative damage by effectively scavenging superoxide anions and attenuating vascular NADPH oxidase-driven superoxide production in vascular smooth muscle cells (VSMC) [57,63,70]. In addition, quercetin is reported to suppress the reduction of NOS activity and increase plasma and urine NO metabolites in SHRs [63,64]. A proposed mechanism is that the radical scavenging properties of quercetin allow for increased bioavailability of endothelium-derived nitric oxide, which enhances the vasodilatory response in blood vessels [54]. Furthermore, oral administration of quercetin is observed to be more effective than intraperitoneal in terms of preventative effects related to cardiovascular complications in SHR [57].

Quercetin is most popular for its blood pressure-lowering and antioxidant properties. However, there is evidence that quercetin also ameliorates structural and functional properties in the heart. The following studies evaluate the effects of quercetin against cardiac hypertrophy in an SHR model. In addition to lower blood pressure and LV-body weight ratio in SHR, Yan et al. reported that quercetin treatment significantly attenuated Ang II-induced H9C2 myoblast cell hypertrophy in vitro. In addition, it suppressed the activation of transcription factors c-fos and s-jun, which are components of activator protein 1 (AP-1), as well as the downstream hypertrophy gene. In addition, the treatment significantly increased peroxisome proliferator-activated receptor γ (PPAR-γ) activity. AP-1 is known to play a significant role in cardiomyocyte hypertrophy. PPAR-γ is a transcription factor that regulates gene expression in the AP-1 pathway and many others. The PPAR-γ dependent pathway has been shown to play a critical role in the inhibition of cardiac hypertrophy [65]. Furthermore, Chen et al. reported that quercetin ameliorated the hypertrophic response such as increased mRNA levels of atrial natriuretic factor and β-myosin heavy chain induced by Ang-II in H9C2 cells. The flavonoid was also able to protect against mitochondrial dysfunction by modulation of the sirtuin 3 /poly (ADP-ribose) polymerase-1 pathway, adding to the protective effects against cardiac hypertrophy [66]. Honcharov et al. reported that quercetin treatment leads to significantly improved morphological and functional parameters of the heart by inhibiting trypsin-like and chymotrypsin-like proteasome activities in the aorta and trypsin-like, and peptidyl-glutamyl peptide-hydrolyzing-like activities in the heart [71]. The proteasome is a dynamic multicatalytic complex that has a large role in protein degradation in most organs. Notably, proteasome function is critical to maintaining the health of the myocardium and modulation of this complex can alter the outcome of many cardiac risk factors and diseases such as high blood pressure, hypertrophy, cardiomyopathy, and ischemic heart disease [72,73]. Therefore, the current literature suggests that quercetin may be able to protect against cardiac hypertrophy in many ways, in addition to its antioxidative and anti-hypertensive effects.

#### 4.1.4. Salt Sensitive Hypertension Models

The Dahl salt-sensitive rat model has been extensively used to study salt-sensitive hypertension and chronic kidney disease, as the kidney plays a critical role in the long-term regulation of blood pressure through the regulation of the body’s fluid and electrolyte balance [74]. In this model, quercetin is reported to significantly reduce the elevated systolic arterial pressure and MAP caused by a high-salt diet, as well as improve kidney function [75,76]. A study by Aoi et al. determined that quercetin reduces epithelial Na^+^ channel (ENaC) mRNA expression in the kidney, but not in the colon, which is significantly increased by a high-salt diet. ENaC is important for the regulation of blood pressure, as it contributes to the reabsorption of Na^+^ in renal tubules [75]. In addition to preventing a rise systolic blood pressure, Makraj et al. reported that quercetin increased urinary output and sodium output, and decreased kidney AT1a mRNA expression in this model [76]. Therefore, quercetin may exert anti-hypertensive effects by protecting the function of the kidneys in the case of salt-sensitive hypertension.

In other salt-induced models of hypertension, quercetin has proved to possess anti-hypertensive effects. In respective comparative studies, quercetin has been reported to be more effective than nifedipine and verapamil, which are calcium channel blockers, in improving hemodynamic and metabolic abnormalities associated with a high salt diet in sodium chloride-induced hypertensive rat and deoxycorticosterone acetate (DOCA)-salt hypertensive rat models [77,78]. In a sodium fluoride-induced hypertension model, quercetin was shown to effectively restore blood pressure, normalize the QRS interval—an electrocardiographic parameter, improve antioxidant defense by improving the expression of heat shock protein 70 and provide cardioprotective effects by increasing the expression of Extracellular signal-regulated kinases (ERK) and PPARγ [79]. Prolonged QRS durations are associated with hypertension-induced LV hypertrophy and are considered an independent risk factor for cardiovascular and all-cause mortality [80]; these results suggest that quercetin is effective in attenuating salt-sensitive hypertension by modulation of the antioxidant defense system.

### 4.2. Clinical Trials

One double-blind, placebo-controlled, crossover design study administered a high dose of quercetin aglycone (1095 mg) to 17 overweight, normotensive, and hypertensive men [81]. Participants in the hypertensive group had an SBP of 142 ± 9 mm Hg and a DBP of 91 ± 7 mm Hg; they were aged 41 ± 12 years with a BMI of 29 ± 5 kg/m^2^. The results of the study determined that quercetin treatment significantly reduced SBP and DBP in the hypertensive group.

Another double-blind, randomized, crossover, placebo-controlled trial studied the effects of quercetin in 49 men aged 48 to 68 years [82]. Participants had a BMI of 26.3 ± 0.3 kg/m^2^ with an SBP of 138.4 ± 2.3 mm Hg and a DBP of 84.4 ± 1.3 mm Hg. There were two groups: *APOE3/3* and *APOE4*. The men received 150 mg/d of quercetin, while the others received the placebo treatment for 8 weeks; those who received the quercetin treatment had their SBP significantly lowered; this beneficial effect was associated with decreased triacylglycerol and increased HDL-C levels.

Egert et al. did a study on how quercetin impacts overweight/obese patients. There were 42 men and 51 women aged 25 to 65 years included in this double-blinded, placebo-controlled, crossover trial. SBP was 130.3 ± 16.4 mm Hg, and DBP was 81.6 ± 9.3 mm Hg, with a BMI of 30.6 ± 3.2 kg/m^2^. For 6 weeks, participants either consumed 150 mg/d of quercetin or a placebo. The results indicated that the *APOE3/3* group that consumed quercetin had their significantly SBP lowered. Participants in the *APOE4* group had no significant effects on blood pressure. The authors suggested that ineffective treatment for the *APOE4* group can be attributed to the fact that carriers of the APOE4 allele may not be responsive to quercetin. The beneficial effect was associated with a decrease in oxidized LDL and TNF-𝜶 in overweight-obese carriers of the apo ε3/ε3 genotype [83].

A similar study looked at how quercetin affects men and women with prehypertension or stage 1 hypertension [84]. For prehypertensives, SBP was 137 ± 2 mm Hg, and DBP were 86 ± 1 mm Hg with a BMI of 29.8 ± 1.3 kg/m^2^. For stage 1 hypertensives, SBP was 148 ± 2 mm Hg and DBP were 96 ± 1 mm Hg with a BMI of 29.3 ± 1.3 kg/m^2^. This randomized, double-blind, placebo-controlled, crossover study had a total of 41 participants who either consumed 730 mg/d of quercetin or a placebo for 12 weeks. For those with stage 1 hypertension, SBP and DBP were significantly lowered. The SBP and DBP of prehypertensive participants were not affected by the quercetin treatment. According to the authors, quercetin’s ability to lower blood pressure may be dependent on the severity of hypertension. Data from preclinical trials support this statement, as decreases in blood pressure due to quercetin supplementation were observed in hypertensive but not normotensive rats [85,86].

Shi and Williamson looked at the effects of quercetin in 22 pre-hyperuricemic men aged 19 to 60 years. Participants had an SBP of 123.2 ± 7.2 mm Hg, and a DBP of 73.8 ± 9.2 mm Hg. The BMI ranged from 18.5 to 29.9 kg/m^2^. This randomized, double-blinded, placebo-controlled, cross-over trial had participants consuming 500 mg of quercetin or a placebo daily for 4 weeks. Quercetin significantly lowered DBP, although there were no changes to SBP [87].

In another double-blind, placebo-controlled, randomized clinical trial, researchers studied how quercetin will affect patients with post-myocardial infarction [88]. There were 88 men and women aged 35 to 65 years included in this study, with a BMI of less than 35 kg/m^2^. For the quercetin group, participants had an SBP of 126.25 (19.41) mm Hg, and a DBP of 10.06 (81.95) mm Hg. For 8 weeks, participants consumed 500 mg/day of quercetin or a placebo. There were no significant changes to SBP or DBP. The authors suggest that this may be due to the anti-hypertensive drugs that these post-MI patients were taking to control hypertension. Therefore, the efficacy of quercetin to lower blood pressure may decrease when the blood pressure is already in a managed state [88].

One double-blinded, randomized, placebo-controlled cross-over trial observed the effects of quercetin in 93 overweight men and women with high-cardiovascular disease risk phenotype [89]. Participants were aged 25 to 65 with a BMI between 25 and 35 kg/m^2^. SBP was 130.3 ± 16.4 mm Hg, and DBP was 81.6 ± 9.3 mm Hg. Participants received 150 mg/d of quercetin or a placebo for 6 weeks. SBP decreased significantly for participants in the quercetin group, with no changes to DBP. The lowering of SBP was associated with the reduction of oxidized LDL levels [89].

The last study looked at the effects of quercetin in patients with gout and essential hypertension [90]. This 12-month clinical trial included 84 men aged 57.2 ± 7.8 years in the main group, and 56.2 ± 6.9 years in the comparative group, with a BMI of 30.9 ± 3.9 kg/m^2^ and 31.1 ± 3.9 kg/m^2^ respectively. The main group participants had an SBP of 137.1 ± 7.9 mm Hg, and a DBP of 84.4 ± 5.5 mm Hg. Comparative group participants had an SBP of 138.8 ± 6.4 mm Hg and a DBP of 84.4 ± 9.7 mm Hg. Participants received 1000 mg of quercetin twice a day for 6 months. Then, the dose was lowered to 500 mg twice a day for another 6 months. The results determined that SBP and DBP decreased significantly. Treatment also lowered uric acid levels and normalized kidney and diastolic heart function in these participants [90].

Overall, the majority of the randomized controlled trials (RCT) outcomes show a significant effect of quercetin supplementation in the lowering of BP, which suggests that this bioactive has the potential to be considered as an add-on to anti-hypertensive therapy. Further well-designed trials are needed to optimize the effective dosage and to investigate the possible drug interactions between quercetin and existing anti-hypertensive medications.

## 5. Resveratrol

Resveratrol is a polyphenol that naturally occurs in a variety of food sources such as grapes, peanuts, apples, plums, blueberries, and soy, with the highest concentrations of resveratrol found in red wine and Itadori tea [91,92]; it has a stilbene structure consisting of two phenolic rings bonded by a double styrene bond which allows for resveratrol to occur in isometric cis- and trans-forms. However, trans-resveratrol is seemingly the most predominant and stable form [93]. Resveratrol is a phytoalexin widely known for its wide range of biological functions, including its antimicrobial, anti-inflammatory, antioxidant, vasorelaxant, neuroprotective, anticarcinogenic, antiviral, and cardioprotective effects, and plays a significant role in maintaining human health [92,93,94,95].

### 5.1. Preclinical Studies

#### 5.1.1. Pulmonary Hypertension Models

Several studies have evaluated the anti-proliferative, antioxidant and anti-inflammatory effects of resveratrol in models of pulmonary hypertension. Resveratrol has been reported to normalize right ventricular systolic pressure and prevent RV hypertrophy, and oxidative stress as well as reduce the expression of inflammatory markers such as IL-6, IL-1, TNF-α, platelet-derived growth factor-α/β, TGF-β, monocyte chemoattractant protein-1 [96,97,98,99,100,101]. Resveratrol also effectively reduces pulmonary vascular remodeling, including medial thickness and muscularization of pulmonary arteries, and PASMCs proliferation [96,97,98,100,101,102,103,104]. In an MCT-induced PAH model, a study by Csiszar et al. reported that resveratrol treatment significantly downregulated NAD(P)H oxidase and improved the expression of endothelial NO synthase (eNOS) which in turn improved endothelial function of the pulmonary arteries [96]. Shi et al. revealed that resveratrol suppressed SphK1/S1P-mediated NF-κB activation and cyclin D1 expression in the MCT-PAH rat model. The SphK1/S1P pathway is known to play an essential role in the development of PAH by activation of NF-κB which then up-regulates the expression of cyclin D1; this leads to PASMCs proliferation and pulmonary vascular remodeling. Lui et al. reported that resveratrol suppressed pulmonary vascular remodeling by modulation of the NR4A3/cyclin D1 pathway [105]. Paffett et al. determined that resveratrol normalized atrophy and apoptosis-mediating pulmonary artery atrogin-1 mRNA expression in MCT-induced pulmonary hypertensive rats [98].

Using a hypoxic pulmonary hypertension (HPH) rat model, Xu et al. reported that resveratrol treatment reduced ROS production and inflammatory markers in PASMCs induced by hypoxic conditions. Resveratrol inhibited the expression of HIF-1α, the main transcriptional regulator of cellular response to hypoxia, by suppression of the MAPK/ERK1 and PI3K/AKT pathways [100]. Resveratrol is thought to interact with this pathway by diminishing the expression and phosphorylation of AKT, therefore preventing the development of HPH [106]. The results of another study determined that resveratrol has an inhibitory effect on arginase II in PASMC, also through modulation of the PI3K/AKT pathway in an HPH rat model [101]. Arginase II metabolizes the conversion of arginine to ornithine and urea; it is known to be important in cell proliferation in various cell types. The induction of arginase II has been shown to play a role in the development of PAH [101]; these results suggest that resveratrol exerts anti-proliferative effects that may attenuate the development of hypoxic pulmonary hypertension.

#### 5.1.2. Renovascular Hypertension Model

A handful of studies have used the 2k1C rat model to determine the anti-hypertensive and cardioprotective effects of resveratrol in the case of renovascular hypertension. In all studies in this category, a consistent finding was significantly reduced systolic blood pressure in 2K1C rats [107,108,109,110,111,112]. Two studies compared the benefits of resveratrol to those of captopril, an angiotensin-converting enzyme inhibitor in the 2K1C model. The results of both studies determined that resveratrol treatment exerted better hypotensive effects than captopril. In one of the studies, resveratrol, alone or in combination with captopril, was able to normalize aortic thickness and reduce aortic fibrosis [111]. In the second study, Resveratrol reduced collagen deposition and whole-heart hypertrophy. A larger reduction in ventricular hypertrophy was observed with resveratrol compared to captopril [110]. In other studies using this model, resveratrol treatment ameliorated contractile responses to phenylephrine [112] and ACh-induced relaxations in the aorta [107] and effectively improved the cardiac hypertrophy index and ROS basal levels in aortic rings [108]. Toklu et al. reported an improvement in parameters related to heart structure and function, such as aortic contractility and left ventricular function. In addition, various oxidative stress markers such as MDA and the activities of glutathione, superoxide dismutase, Na^+^/K^+^-ATPase, lactate dehydrogenase and catalase were markedly improved [112]. In a diabetic renovascular hypertension study, resveratrol was able to lower systolic blood pressure, improve glucose and lipid metabolism and improve the expression of enzymes in the antioxidant defense system [109]; these results provide evidence that resveratrol is effective in combating oxidative stress, hypertension, and hypertrophy and in the event of renovascular hypertension.

#### 5.1.3. SHR Models

A large body of research has analyzed the efficacy of resveratrol in improving cardiac and metabolic parameters in an SHR model. Many studies have observed attenuation of high blood pressure in this model following resveratrol treatment [15,113,114,115,116,117], while others did not see an improvement [118,119,120,121,122]. In addition, resveratrol has been reported to improve a multitude of parameters related to oxidative stress, hemodynamic parameters, and cardiac morphology in this model. The cardio-protective and anti-hypertensive effects of resveratrol are observed to be much more pronounced in SHR rather than in their Wistar Kyoto normotensive controls. Chronic resveratrol treatment was shown to significantly improve aortic endothelium-dependent relaxation to acetylcholine [115,117,119] and endothelium-independent relaxation to sodium nitroprusside [117]. Resveratrol also effectively improves NO bioavailability, increases eNOS and AMPK activities and prevent eNOS uncoupling in SHRs, which would result in improved endothelial function and vasodilation [113,114,115,117,118,123,124]. Furthermore, uncoupled eNOS generates ROS, which would reduce NO bioavailability and contribute further to oxidative stress.

Many studies have observed the antioxidant effects of resveratrol in this model. In vitro studies following resveratrol treatment have shown attenuation of an array of oxidative stress markers such as superoxide anion [125], H_2_O_2_ [117,119,126], nitrotyrosine [116,117], 8-isoprostane [15,116], protein carbonyl [15] and TBARS [113,116,118,120,121,122]. In addition, it has also effectively improved the activity of enzymes in the antioxidant defense system such as superoxide dismutase [15,113,117], glutathione peroxidase [113], glutathione reductase [113], glutathione-S-transferase [15] and catalase [126], and reduced NAD(P)H reductase, heme oxygenase-1, NAD(P):quinine oxidoreductase-1 activities [116,125]. Moreover, resveratrol treatment was shown to decrease the infiltration of pro-inflammatory immune cells such as lymphocytes, macrophages, and Ang II-positive cells in renal tubulointerstitial areas, which may suggest the polyphenolic compound may also possess anti-inflammatory qualities [15].

In terms of cardiac function, resveratrol has proven to be effective in preventing the development of hypertrophy and cardiac dysfunction. Grujic-Milanovic et al. reported resveratrol treatment provided protective effects against hypertrophy by preserving the lamina elastica interna and elastic fibers in the aortic endothelium of female SHR and blunting the expression of TGF-β, which mediates the promotion of vessel structure alterations [113]. Thandapilly et al. observed prevention in the increase of the diastolic functional parameter, IVRt, and an improvement in the systolic functional parameter ejection fraction in 20-week-old SHR [122]. Another study by the same laboratory group determined that resveratrol reduced collagen deposition in left ventricular tissue and prevented a reduction in fractional shortening as well as attenuated stiffening of wall components of SHR arteries [120]; these results suggest resveratrol can prevent the development of concentric hypertrophy and contractile dysfunction. Another study reported reduced vascular remodeling and also attenuated ERK signaling and the expression of proliferating cell nuclear antigen in arteries of SHR following resveratrol treatment [121]. Other anti-proliferative effects of resveratrol such as attenuation of the overexpression of cell proliferation proteins such as cyclin D1, cyclin E, Cdk2/4, phosphorylated retinoblastoma protein, Giα proteins and enhanced phosphorylation of ERK1/2 and AKT in SHR VSMC have been observed; this effectively lessened the enhanced proliferation of VSMC which is a complication of persistent hypertension [125].

#### 5.1.4. Salt-sensitive Hypertension Models

Few studies have evaluated the effects of resveratrol in salt-sensitive models. Using a Dahl salt-sensitive hypertensive rat model, Rimbaud et al. observed a significant improvement in fraction shortening and systolic, diastolic, and aortic endothelial functions were preserved without any changes in blood pressure following resveratrol treatment [127]. In a DOCA salt induced hypertension model, attenuation of systolic blood pressure has been observed in a few studies [128,129,130]. In addition to improved acetylcholine-induced endothelium-dependent relaxation and antioxidant status, Han et al. reported that resveratrol modified histone 3 lysine 27 methylation in the aorta and renal artery of DOCA rats [129]. This result suggests that resveratrol may improve cardiac function through epigenetic modification. Sun et al. evaluated the effects of resveratrol treatment in AMPKα2^−/−^ DOCA mice. The results of the study determined that the loss of AMPKα2 function reversed the vasodilatory effects of resveratrol, suggesting that resveratrol’s anti-hypertensive effects rely heavily on its activation of AMPK [130]. In another study, cardiac parameters such as left ventricular wet weight, left ventricular wall thickness, diastolic stiffness constant and cardiac contractility were improved in DOCA rats treated with resveratrol. In addition, resveratrol decreased inflammatory cell infiltration and cardiac fibrosis as well as improved cardiac and vascular function [128]. Though more studies are needed to determine the effects of resveratrol treatment in the case of salt-sensitive hypertension.

### 5.2. Clinical Trials

Resveratrol can control blood pressure, oxidative stress, cell adhesion, oxidative stress, and endothelial function [131]. Numerous human interventional studies have examined the significant benefits of resveratrol on the blood pressure of hypertensive individuals to date. In one double-blind, randomized, placebo-controlled trial, the effects of resveratrol in type 2 diabetic patients were examined [132]. Participants were overweight, with a BMI which ranged between 28.2 and 29.5 kg/m^2^. A total of 192 men and women aged 40 years or older were included in this 6-month study with SBP between 131.8 and 134.1 mm Hg, and DBP between 80.9 and 81.4 mm Hg. There were three groups for this study: the first group received 500 mg of resveratrol a day, while the second group received 40 mg of resveratrol per day, and the last group received the placebo. There were no significant changes to blood pressure in either of the three groups. The authors speculate that this might be due to the study’s limited sample size, which might have prevented resveratrol from having its full potential [132].

In the second study, a prospective, open label, randomized, controlled trial was done on 62 men and women aged between 30 to 70 years with type 2 diabetes [133]. Participants in the intervention group had a SBP of 139.71 ± 16.10 mm Hg and a DBP of 81.42 ± 9.58 mm Hg; these individuals had a BMI of 24.66 ± 3.62 kg/m^2^. Participants received 250 mg of resveratrol per day with oral hypoglycemic agents, while the control group received only oral hypoglycemic agents for 3 months, respectively. The results showed that there were significant reductions in SBP and DBP in the individuals who consumed resveratrol daily. This favorable effect was linked to a reduction in LDL-C and an improvement in glycemic index [133].

Another randomized, double-blind, placebo-controlled clinical study observed how resveratrol affects the blood pressure of individuals who are overweight/obese and have non-alcoholic fatty liver disease [134]. The study was conducted on 50 males and females aged between 20 and 60 years with a BMI between 25 and 35 kg/m^2^. SBP ranged between 131.61 ± 17.97 mm Hg, and DBP ranged between 85.30 ± 13.70 mm Hg. Individuals in the placebo group received corn starch capsules, while participants in the intervention group received 600 mg of resveratrol daily for 12 weeks. There were no significant results on SBP or DBP. The authors state that the number of clinical disturbances before the administration of resveratrol may have affected the blood pressure results [134].

A randomized, double-blinded, placebo-controlled, parallel-group trial was conducted to observe the effects of resveratrol in 24 obese men ages 18 to 70 years for 4 weeks [135]. Individuals had a SBP of 124.3 ± 2.9 mm Hg and a DBP of 75.6 ± 2.1 mm Hg; these men had a BMI greater than 30 kg/m^2^ and received 500 mg of *trans*-resveratrol per day. The results proved that there were no significant effects on SBP or DBP of the participants. According to the authors, this may be attributed to the study’s short duration or the baseline characteristics of those who participated in this trial [135].

In a similar study, Marques et al. evaluated the effects that acute trans-resveratrol had on individuals with endothelial dysfunction in a randomized, cross-over, double-blind, placebo-controlled trial [136]. There were 24 participants between the ages of 45 and 65, which included both males and females. BMI of these individuals was 30 ± 1 kg/m^2^. SBP ranged between 139 ± 2 and 142 ± 2 mm Hg, and DBP was 87 ± 2 mm Hg. Patients in the intervention group received 300 mg/day. According to the results, there were no changes to SBP and DBP in the intervention group, which may be due to the small sample size they used in this study [136].

The effect of resveratrol in 11 obese men aged 52.5 ± 2.1 years were observed in a randomized double-blind crossover study [137]. SBP ranged between 132 ± 3.0 mm Hg, and DBP ranged between 83 ± 2.6 mm Hg. BMI was between 31.45 ± 0.82 kg/m^2^. For 30 days, individuals in the intervention group consumed 150 mg of resveratrol per day, while the others received the placebo treatment. There were significant reductions in SBP for those who consumed the resveratrol capsules. Reduced glucose levels, triglyceride levels, and inflammation markers were linked to this positive effect [137].

Kjær et al., studied how resveratrol affects patients with metabolic syndrome in a randomized, placebo-controlled, double-blind, parallel-group clinical trial [138]. There were 74 men aged between 30 and 60 years included in this study. SBP ranged between 140 ± 2.34 and 150 ± 3.44 mm Hg, and DBP between 86.9 ± 1.54 and 91.3 ± 2.10 mm Hg. Participants were obese, with a BMI between 33.4 ± 0.858 and 34.1 ± 0.770 kg/m^2^. For 16 weeks, participants either received a high dose of resveratrol (1000 mg/day), a low dose of resveratrol (150 mg/day), or a placebo treatment. There were no significant changes to SBP or DBP in either group. The study’s design, including the dosages administered, the duration of the study, and the criteria for participants to be included in this study, lead the authors to speculate that there was no effect on blood pressure [138].

Another study observed the effects of resveratrol in 41 overweight/obese men and women in a parallel-group, double-blind clinical trial [139]. Participants were aged 40 to 70 years with a BMI between 27 and 35 kg/m^2^. SBP ranged between 132 ± 2.2 and 135 ± 3.5 mm Hg and DBP ranged between 83 ± 1.7 and 88 ± 2.2 mm Hg. For six months, individuals consumed 150 mg/d of resveratrol, or the placebo treatment. There were no significant effects on SBP or DBP. According to the authors, there are no known mechanisms linking resveratrol to changes in blood pressure [139].

There are conflicting findings in the human studies that examined how resveratrol affects hypertensive patients. Despite the fact that some trials had no impact on blood pressure, many of them showed a reduction in SBP and DBP. The anti-hypertensive effects that this bioactive provides are anticipated to have more favorable results with further human trials that have wider inclusion criteria and populations, as well as higher doses of resveratrol when conducting the study.

## 6. Coenzyme Q10

Coenzyme Q10 (CoQ_10_) is a 1,4-benzoquinone, also known as ubiquinone [45]; it is a lipid-soluble compound mainly found in organ meats such as pork and beef liver, oily fish including salmon and tuna, whole grains, fruits and vegetables such as spinach and broccoli, blackcurrants and strawberries, and yogurt and cheese [140,141]; it is particularly known to aid in the prevention of inflammation and cardiovascular disease due to its antioxidant properties [142]. Additionally, it may help in the prevention of hypertension, obesity, AIDS, diabetes, kidney failure, gastric ulcers, Parkinson’s disease, headaches, aging, and mitochondrial disorders [141]. CoQ_10_ is also essential for the production of cellular energy and is known to play a role in immune function [140.

### 6.1. Preclinical Studies

There are a very limited amount of recent pre-clinical trials evaluating the effects of coenzyme q10 in the models previously described. In an SHR model, CoQ_10_ treatment has been reported to reduce myocardial hypertrophy, improve antioxidant defense, and reduce the generation of ROS in the cardiac mitochondria, though no improvements in blood pressure were observed [143]. Using an SHR/cp model, which is commonly used as a model for metabolic syndrome, CoQ_10_ treatment prevented a rise in blood pressure and serum insulin levels and improved endothelial dysfunction in mesenteric arteries. In addition, oxidative and nitrative stress markers and inflammatory markers were improved in a dose-dependent manner [144]. In stroke-prone SHRs, CoQ_10_ attenuated a rise in blood pressure and prevented renal membranous phospholipid degradation; it also enhanced phospholipase A activity. This suggests that CoQ_10_ may have a protective effect against structural and functional dysfunction in renal cells, which may also protect against the development of renovascular hypertension [145]. Analogues of CoQ_10_ such as decylubiquinone and MitoQ_10_, a mitochondria-targeted ubiquinone, have proven to be effective as a therapeutic intervention in stroke-prone SHRs, as they both effectively reduced systolic blood pressure. In addition, decylubiquinone significantly improved plasma MDA levels and markers of lipid metabolism [146]. MitoQ_10_ was reported to attenuate aortic superoxide production, improve NO bioavailability, and significantly reduce cardiac hypertrophy compared to the control. As most conventional antioxidants cannot penetrate the mitochondria, MitoQ10 may provide a novel therapeutic approach to target oxidative stress specifically generated by mitochondria [144]. In a 2K1C model, a poly(lactide-co-gylcolide) nanoparticulate formulation of CoQ_10_ was reported to considerably improve the efficacy of the antioxidant. Systolic and diastolic blood pressure and MDA were significantly improved in 2K1C rats [147]. Ubiquinol, the reduced form of CoQ_10_ was shown to also lower blood pressure, improve renal superoxide production and decrease urinary albumin levels salt-induced hypertension rat model [148]. More pre-clinical trials are needed to determine the anti-hypertensive effects and mechanistic actions of CoQ_10_.

### 6.2. Clinical Trials

Coenzyme Q10 is an antioxidant that is well-known for fighting free radicals and the harm they cause, particularly to the cardiovascular system [141]. There has been a significant number of human trials that investigated the effects of CoQ_10_ in hypertensive individuals. One study observed the effects of CoQ_10_on 109 Caucasian/African American men and women aged 27 to 89 [149]. Participants had a SBP of 159 mm Hg, and DBP of 94 mm Hg. For 13 months, participants consumed 225 mg of CoQ_10_ or a placebo daily. For those who consumed coenzyme Q10, both SBP and DBP decreased significantly, and diastolic heart function was improved.

The impact of CoQ_10_ was assessed in 64 males with coronary artery disease in a different randomized, double-blind trial [150]. Participants were aged 48.3 ± 7.2 years in the CoQ_10_ group, with a BMI of 23.9 ± 1.2 kg/m^2^. SBP was 168 ± 9.6 mm Hg and DBP was 106 ± 4.6 mm Hg. Participants received 120 mg/d of coenzyme Q10 or a placebo for 8 weeks. The results indicated that there were significant reductions in SBP and DBP for those who consumed the CoQ_10_ treatment; these beneficial effects were associated with reductions in oxidative stress, triglycerides, and insulin, and an increase in HDL-C [150].

Another randomized, double-blind, placebo-controlled 12-week crossover trial studied how CoQ_10_ affects individuals with metabolic syndrome [151]. Participants included 30 obese men and women aged 25 to 75 years with a BMI of 32.1 ± 0.9 kg/m^2^. SBP was 147.8 ± 2.1 mm Hg, and DBP was 77.4 ± 2.2 mm Hg. For 12 weeks, participants received 200 mg/day of coenzyme Q10 or a placebo. There were no changes in SBP or DBP. The authors suggest that patients with higher baseline BP may have needed to be included in this study in order to see significant changes in BP [151].

One study examined the impact of CoQ_10_ on 74 men and women with dyslipidemia and type 2 diabetes [152]. Participants were aged 31 to 75 years, with a BMI less than 40 kg/m^2^. Patients in the CoQ_10_ group had a SBP of 127.1 mm Hg, and a DBP of 75.5 mm Hg. For 12 weeks, patients received a daily dose of 200 mg of CoQ_10_ or a placebo in this randomized double-blind, placebo-controlled intervention. CoQ_10_ significantly lowered SBP and DBP; this decrease in blood pressure was linked with improved long-term glycaemic control in type 2 diabetes patients [152].

The last study examined how CoQ_10_ affects type 2 diabetic patients [153]. 74 overweight patients aged 40 to 79 years were included in this randomized, double-blind trial, with a BMI of 28.7 ± 3.4 kg/m^2^. SBP was 132.8 ± 17.3 mm Hg, and DBP was 74.1 ± 9.2 mm Hg. For 6 months, participants either received 160 mg of fenofibrate, 200 mg of CoQ_10_, 160 mg of fenofibrate and 200 mg of CoQ_10_, or a placebo. Fenofibrate and CoQ_10_ independently decreased DBP and, in combination, also significantly reduced SBP [153].

Overall, the research to date has shown promising findings that support the anti-hypertensive properties of CoQ_10_. We may draw the conclusion that CoQ_10_ can most likely function as an anti-hypertensive bioactive for patients with high blood pressure based on the human trials that are currently available.

## 7. DHA and EPA

DHA is a long chain n-3 polyunsaturated fatty acid (PUFA), abundantly found in fish oil [154]. Additionally, it is present in eggs, meat, poultry, marine algae, and breast milk [155,156]. DHA is essential for optimal fetal development and a healthy cardiovascular system [157]. DHA consumption provides vasoprotective, antioxidant, and anti-inflammatory effects (Figure 3) [158].

Eicosapentaenoic acid is also long chain n-3 PUFA [159]; it mainly derives from fish and fish oil [157,160]. Foods with high omega-3 content include nuts, cold-water fatty fish, seeds, leafy vegetables, and vegetable oil [161,162]. As an n-3 fatty acid, EPA has been proven to have anti-inflammatory, anti-hypertensive, antithrombotic, and triglyceride-lowering effects [157,163,164] (Figure 3). There is significant evidence that EPA aids in the management of dementia, coronary heart disease, depression, and rheumatoid arthritis [159].

### 7.1. Preclinical Studies

#### 7.1.1. Pulmonary Hypertension Models

The protective effects of DHA and EPA have been evaluated in different models of pulmonary hypertension. In MCT-induced PAH rat models, DHA decreased mPAP and reduced pulmonary vascular remodeling and RV hypertrophy; it also improved lung inflammation and suppressed the accumulation of macrophages and T lymphocytes in lung and pulmonary arteries. Additionally, DHA may provide anti-proliferative effects by arresting the cell cycle of PASMCs through the inhibition nuclear factor of activated T cells-1 [165]. In another study, DHA decreased NF-κB and p38 MAPK activation, which led to a reduction in vascular endothelial growth factor and biomarkers of PAH, MMP-2, MMP-9 [166]. Using a hypoxic pulmonary hypertension rat model, DHA is reported to reduce right ventricular systolic pressure and improve right ventricular hypertrophy [167]; it also prevents proliferation, migration and phenotype switching of PASMCs induced by hypoxia [167].

In MCT-induced PAH rats, EPA is reported to lower systolic pulmonary arterial pressure, provide anti-inflammatory effects by improving pulmonary GPR120 mRNA expression and inhibit PASMC proliferation stimulated with TGF-β or FGF_2_ [168]. In addition, EPA was shown to ameliorate vascular remodeling and vasoconstriction and suppress vasoconstriction, probably by the downregulation of SRC family kinases in the same model [169]. EPA treatment may also be able to prevent pulmonary edema by decreasing arachidonic acid content and LTB4 generation and plasma thromboxane B2 in rats with endotoxin-induced lung injury [170]. In sheep pulmonary arteries, EPA supplementation stimulated NO release and improved endothelium-dependent and -independent relaxations, possibly through modulation of Ca^2+^ influx through L-type calcium channels [171].

#### 7.1.2. SHR Model

A handful of studies have assessed the anti-hypertensive and cardio-protective effects of DHA supplementation (not in combination with other essential fatty acids) in an SHR model. DHA supplementation alone can effectively prevent a rise in blood pressure in SHR [172,173,174,175].

In addition, it has been reported to reduce vascular wall thicknesses in the coronary, thoracic, and abdominal aorta. DHA was able to induce relaxant effects in both norepinephrine and high-K^+^-induced contracted SHR aortic rings but not improve aortic contractile or relaxant responses to acetylcholine or nitroprusside, which suggests that the vasorelaxant effects of DHA are associated with intracellular Ca^2+^ release and modulation of L-type Ca^2+^ channels in VSMC and are independent of endothelium-derived nitric oxide [175,176]. A couple of studies have observed blood pressure lowering effects from DHA, but not EPA supplementation in SHR [173,174] In addition, Rousseau-Ralliard et al. reported an improvement in the electrocardiographic parameter QT, most likely by preferential incorporation of DHA into cardiac tissues and by influencing beta-adrenergic function [174]. Other studies have reported that DHA supplementation altered hepatic DHA, EPA, and n−6 fatty acid content, decreased hepatic delta-9-desaturase activity, reduced aldosterone and corticosterone levels as well as modulated lipid metabolism in SHR [172,177,178]

Past studies have shown that EPA supplementation can provide anti-hypertensive effects in SHR. A couple of studies found EPA treatment reduced systolic blood pressure in this model [179,180], while others found no improvements [173,174,181,182]. EPA treatment was found to reduce serum thromboxane B2 levels, induce endothelium dependent relaxations in norepinephrine-contracted aortic rings [182] and improve those induced by acetylcholine but not endothelium-independent relaxations to sodium nitroprusside [181]. EPA prevented exaggerated growth in SHR VSMC cells by suppressingTGF-β expression, reduced CDK2 activity and inhibiting basal DNA synthesis [183]; these results suggest that EPA is able to improve vascular reactivity and inhibit exaggerated cell proliferation which may prevent the development of hypertension in SHR.

### 7.2. DHA Clinical Trials

There has been sufficient evidence to suggest that DHA contributes to many functions of the body, particularly cardiovascular function. Currently, there are only three studies that have assessed the effects of DHA on blood pressure in hypertensive individuals. In a study by Sagara et al., 156 Scottish men aged 45 to 59 who were overweight, hypertensive, and hypercholesteremic were included [184]. Participants in the DHA group had a SBP of 141.4 ± 13.5 mm Hg, a DBP of 86.7 ± 10.4 mm Hg, and a BMI of 26.4 ± 3.3 kg/m^2^. For five weeks, men in the DHA group consumed 2 g of DHA per day, while the placebo group received 1 g of olive oil per day. The outcomes showed significant reductions in the SBP and DBP of the participants in the DHA intervention group when compared to the placebo group. The beneficial effect of DHA could be associated with a lowering of LDL-C and an increase in HDL-C [184].

The second study was a double-blind, placebo-controlled trial of parallel design done on 39 participants, which included men and postmenopausal women ages 40 to 75 years [185]. This study examined the effects of both DHA and EPA on treated hypertensive individuals with type 2 diabetes. Participants were categorized as overweight to obese according to their BMI, which ranged from 27.9 to 30.6 kg/m^2^. SBP ranged between 115 to 180 mm Hg, and DBP was less than 110 mm Hg. There were three groups: the placebo group, the DHA group, and the EPA group. Each participant consumed 4 g per day in their respective group for 6 weeks. The findings revealed that neither DHA nor EPA had any significant effects on blood pressure. The authors have suggested a number of potential explanations for why there were no changes in blood pressure, including the use of other pharmaceutical treatments, adequacy of glycemic control, higher variability in blood pressure in diabetes patients, insufficient statistical power, and the use of olive oil as a placebo [185].

The last study looked at how DHA and EPA impacted the blood pressure in 59 mildly hyperlipidemic, overweight men. Participants were ages 20 to 65 years old with a BMI between 25 to 30 kg/m^2^ [186]. SBP was between 119.1 and 124.2 mm Hg, and DBP between 71.4 and 75.2 mm Hg. In this double-blind, placebo-controlled trial of parallel design, participants received 4 g/d of placebo, EPA, or DHA for 6 weeks. The results showed that individuals in the DHA group had their SBP and DBP lowered significantly. There were no significant changes to BP for those in the EPA group [186].

Overall, the number of human studies that have assessed the direct effects of DHA on hypertension is very limited. Two out of the three human trials show that DHA independently lowers SBP and DBP. More human trials need to be conducted in order to affirm these effects in hypertensive patients.

### 7.3. EPA Clinical Trials

EPA has often been a recommended source to prevent cardiovascular disease, and all complications related to the cardiovascular system [159]. There are a limited number of human trials done on the anti-hypertensive effects of EPA on hypertensive patients. The effectiveness of EPA was studied in untreated patients with hyperlipidemia [187]. Participants included 24 men and women aged 58 ± 6 years. For those in the EPA group, BMI was 21.2 ± 2.3 kg/m^2^. SBP was 141.2 ± 11.1 mm Hg, and DBP was 86.8 ± 6.4 mm Hg. For 3 months, patients either received 1800 mg/d of EPA or 10 mg/d of pravastatin. SBP and DBP significantly decreased in the EPA group; this beneficial effect was associated with a reduction of total cholesterol [187].

Another study looked at how EPA affects the blood pressure of mild to moderate essential hypertensive patients [188]. There were 17 participants, and those in the EPA group had a SBP of 152.9 ± 17.3 mm Hg and a DBP of 99.1 ± 13.8 mm Hg. For 8 weeks, participants either consumed 2.7 g/day of EPA or placebo. SBP significantly decreased for participants who consumed EPA. The beneficial effect of EPA was associated with a reduction in the intracellular concentration of sodium in erythrocytes. There was no effect on DBP. The authors speculate that the reason there was no effect on DBP may be due to the low dosage of EPA administered and the small population included in this study.

In a similar study, a double-blind, placebo-controlled trial of parallel design was done on 39 postmenopausal women and men to study the effect of EPA and DHA on blood pressure [185]. Participants were aged 40 to 75 with a SBP between 128 and 133 mm Hg, and a DBP between 70 to 73 mm Hg. Participants had type-2 diabetes and were overweight/obese with a BMI between 27.9 and 30.6 kg/m^2^. Individuals consumed 4g of EPA, DHA, or a placebo for 6 weeks. There were no significant changes to the SBP or DBP of the participants. The use of supplemental drugs for treatment, adequate glycemic control, the higher changes in blood pressure in diabetes patients, using olive oil as a placebo and a lack of statistical power are possible explanations put forth by the authors as to why there were no changes in blood pressure [185].

Overall, the studies so far show mixed results about the role that EPA plays in lowering blood pressure for hypertensive patients. More robust studies with increased sample sizes, higher EPA doses, and broader population criteria are needed to validate the clinical benefits of EPA.

## 8. Safety and Adverse Effects of the Compounds

### 8.1. Quercetin

Purified quercetin is available as a dietary supplement in doses of up to 1000 mg/day [189]. According to findings from human intervention trials, supplemental quercetin intake is well-tolerated, and the Incidence of adverse effects is very low [189]. Studies that evaluated the safety of quercetin administration in humans included a wide range of doses, ranging from 150–2000 mg/day [84,89,190,191]. Quercetin was safe and well-tolerated at all levels. It is worth noting that only the effect of short-term Quercetin intake (a maximum duration of 12 weeks) was reported in these studies. Furthermore, only a few studies reported detailed information on the safety of quercetin in different disease conditions [189].

### 8.2. Resveratrol

Resveratrol is generally well-tolerated; however, gastrointestinal, and nephrotoxic adverse effects have been documented in humans [135,192,193]. The safety of different doses of resveratrol was evaluated in human trials. Resveratrol was reported to be safe for a 60-kg individual when administered at a dose of 450 mg/d. Higher doses of resveratrol (>1000 mg/d) were reported to inhibit cytochrome P450 isoenzymes; therefore, interacting with many other medications [193,194]. At a dosage of 1000 mg/d, resveratrol was reported to elevate CVD biomarkers such as oxidized low-density lipoprotein, total plasminogen activator inhibitor, and soluble vascular cell adhesion molecule-1 in overweight subjects [195].

### 8.3. Coenzyme Q10

Data from clinical trials indicate that coenzyme Q10 has a very good safety profile; it does not accumulate in plasma or tissues after the termination of supplementation [196]. In healthy adults, coenzyme Q10 administration for 4 weeks at 300, 600, and 900 mg/d was reported to be safe and well-tolerated [196]. In addition, no serious adverse effects were reported when coenzyme Q10 was taken by patients with Parkinson’s disease at 1200 and 2400 mg/d [197]. Common adverse effects associated with coenzyme Q10 intake include gastrointestinal adverse effects and common cold symptoms [196].

### 8.4. EPA and DHA

The safety and tolerability of prescription omega-3 fatty acid products (EPA and DHA) was evaluated in a meta-analysis of 21 randomized controlled trials [198]. There was no serious adverse effects associated with EPA and DHA intake (*n* = 12,750 participants) compared with the control group (*n* = 11,710 participants). Treatment with omega-3 fatty acids was associated with dygeusia, gastrointestinal disorders, and skin abnormalities (eczema, itching, and eruption). In addition, some adverse effects such as gastrointestinal disturbance and low-density lipoprotein cholesterol elevation were more pronounced with EPA/DHA combination products [198].

## 9. Conclusions and Future Directions

In summary, all the bioactive compounds discussed in this review have shown strong evidence of blood pressure-lowering effects in animal and human studies. The perceived anti-hypertensive effects can range from antioxidant effects to modifying signal transduction in vascular physiology. Nevertheless, certain limitations of the current review need to be considered as these studies show some level of heterogeneity, making it difficult to draw concrete conclusions in relation to types of subjects, dosage, form of analyzed product or interactions with other disease conditions. Future studies must try to avoid the risk of potential bias associated with other confounding factors such as other dietary or lifestyle factors.

The knowledge of the anti-hypertensive effects, bio-availability and the most effective dosage of these compounds may be useful information for (1) Plant breeders to create new cultivars with high levels of these compounds using conventional breeding in conjunction with emerging molecular biology techniques (2) primary processors to develop pre and post harvesting strategies to optimize the levels of these compounds (3) Food technologists to enhance the levels of these compounds through processing technologies to deliver required quantities in appropriate servings. The information provided in this review is a step towards the goal of finding a dietary solution for preventing or managing hypertension and its complications

Future clinical studies should focus on the study design to eliminate the risk of bias related to potential confounding variables such as other underlying conditions or lifestyle factors. Moreover, the dosages, source, length of intervention and frequency of consumption should be considered. In order to take these molecules into mainstream clinical usage, it is necessary to carry out better quality RCTs (crossover design, double-blinded, long-term, placebo/controlled) with advanced and accurate outcome measures (such as ambulatory blood pressure monitoring). Further interventional trials are necessary to determine the clinical value of supplementation and to identify possible drug interactions between these bioactive molecules and standard anti-hypertensive medications. For instance, there have been studies showing that quercetin can be metabolized by the cytochrome P450 system [199].

Overall evidence from a large body of preclinical and human studies suggests that the bioactive compounds discussed in this review (DHA, EPA, resveratrol, quercetin, and coenzyme Q10) could have important and clinically relevant anti-hypertensive effects. However, well-designed clinical trials are still required to o widen our understanding towards the underlying mechanisms of action and to narrow the knowledge gap between preclinical findings and human trial data.

## Data Availability

Not applicable.
